# SbSI Composites Based on Epoxy Resin and Cellulose for Energy Harvesting and Sensors—The Influence of SBSI Nanowires Conglomeration on Piezoelectric Properties

**DOI:** 10.3390/ma13040902

**Published:** 2020-02-18

**Authors:** Bartłomiej Toroń, Piotr Szperlich, Mateusz Kozioł

**Affiliations:** 1 Institute of Physics–Center for Science and Education, Silesian University of Technology, ul. Krasińskiego 8, 40-019 Katowice, Poland; bartlomiej.toron@polsl.pl (B.T.); piotr.szperlich@polsl.pl (P.S.); 2 Faculty of Materials Engineering, Silesian University of Technology, ul. Krasińskiego 8, 40-019 Katowice, Poland

**Keywords:** SbSI nanowires, nanocomposite, nanogenerator, nanosensor, energy harvesting, FRP laminate

## Abstract

In this paper, ferroelectric antimony sulfoiodide (SbSI) nanowires have been used to produce composites for device fabrication, which can be used for energy harvesting and sensors. SbSI is a very useful material for nanogenerators and nanosensors in which the high values of the piezoelectric coefficient (d_33_ = 650 pC/N) and the electromechanical coefficient (k_33_ = 0.9) are essential. Alternatively, cellulose and epoxy resin were matrix materials in these composites, whereas SbSI nanowires fill the matrix. Piezoelectric response induced by vibrations has been presented. Then, a composite with an epoxy resin has been used as an element to construct a fiber-reinforced polymer piezoelectric sensor. For the first time, comparison of piezoelectric properties of cellulose/SbSI and epoxy resin/SbSI nanocomposite has been presented. The influence of concentration of SbSI nanowires for properties of epoxy resin/SbSI nanocomposite and in a fiber-reinforced polymer based on them has also been shown. Results of aligning the SbSI nanowires in the epoxy matrix during a curing process have been presented as well.

## 1. Introduction

Nowadays, the demand for different types of energy harvesting increases. Among them are thermal, solar, chemical, nuclear, and mechanical energies. Currently, the pyroelectric [1,2], triboelectric [3,4], and piezoelectric [5,6] effects are frequently used to convert appropriate energy types into electrical energy. The most common energy source available is mechanical energy, and the piezoelectric effect is widely used to harvest it. The first mention of a piezoelectric generator based on ZnO nanowires to convert mechanical energy to electrical energy is in 2006 [6]. Since then, many investigations have been performed on piezoelectric nanogenerators, to harvest, e.g., vibrations, wind, human body motion, wind, acoustic waves, and other mechanical energy forms [7,8,9,10]. Nowadays, various materials and their composites especially ZnO [6,11,12,13,14], BaTiO_3_ [15,16,17], PZT [18], and many other compounds [18] are used to construct piezoelectric nanogenerators. Epoxy resins [12,17] or different types of cellulose [13,14,15,16] are used as a matrix material in these composites.

The most common method to induce voltage generation is the use of mechanical vibrations [19]. This energy form can be easily found in everyday life, i.e., household appliances, vehicle movement, etc. The most typical vibrations with the highest amplitudes occur every day for frequencies lower than 100 Hz. To use these energy sources in the most efficient way, it is essential to construct devices to convert vibration energy within this frequency range. Smart textiles and wearable nanogenerators are other branches in which piezoelectric and triboelectric nanogenerators may be successfully and efficiently use. They may be fabricated based on rayon, cotton, or wool [20,21,22], as well on SbSI nanowires [23]. Recently, there is a growing demand for these kinds of energy sources. They should allow converting the energy of human movement into the electrical energy to supply and charge mobile electronics in the future. Electrical, mechanical, and thermal properties were measured for textiles coated with nanomaterials and were found to be important for the performance of smart textiles [24].

Piezoelectric materials can also be used as integrated sensors [19,25], e.g., in composite laminates that are the basic advanced construction materials. They are commonly used to measure strain and deformation in constructions. It allows monitoring of the material condition to prevent its destruction progress, e.g., airplanes, ships, and bridges.

Recently, there is a growing interest in nanogenerators and nanosensors which are fabricated based on SbSI nanowires [23,26,27,28,29,30,31]. SbSI is a semiconductor and ferroelectric material exhibits very good electromechanical (k_33_ = 0.9) [32] and piezoelectric (d_33_ = 650 pC/N) properties [33]. Because of the huge values of the mentioned parameters, sensors based on SbSI are competitive to sensors made of other materials.

In recent years, a fabrication technology of cellulose/SbSI nanowires composite (CSNC) [29] and epoxy resin/nanowire composite (ESNC) [26] has been published. In this article, we have briefly described the similarities and differences in the case of both processes and the further use of ESNC to produce a fiber-reinforced polymer (FRP) laminate sensor [31]. For the first time, results of piezoelectric response under vibration and their comparison for CSNC and ESNC have been presented in this article. The influence of concentration of SbSI nanowires for properties of ESNC and in a fiber-reinforced polymer based on them has also been shown. Results of preliminary research of aligning the SbSI nanowires in an epoxy resin matrix during a curing process in an electric field have been presented.

## 2. Materials and Experimental Methods

**Figure 1:**
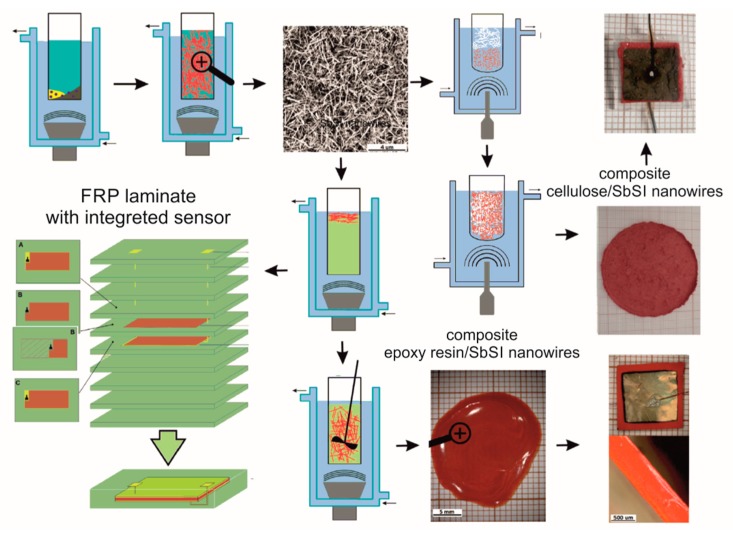
Scheme of sonochemical preparation of SbSI nanowires, the epoxy resin/ SbSI nanowire and cellulose/SbSI nanowires composites, generator/sensor assembly, and scheme of FRP laminate with integrated sensor [26,29,31]. Description in the text.

Fabrication of CSNC, ESNC, and FRP laminates based on ESNC is summarized in Figure 1. The first stage in the preparation of both composites and the FRP sensor is the sonochemical fabrication of SbSI nanowires by the so-called Nowak’s method. Pure elements (antimony, sulfur, and iodine) were used to sonochemically obtain SbSI nanowires. The stoichiometric mixture of elements was placed in ethanol in a closed container made of polypropylene. It did not allow volatile synthesis products to escape. After that, the container was submerged in water in a cup-horn connected to ultrasonic processor VCX-750 with converter VC-334 (Sonics & Materials, Inc., Newtown, CT, USA). Refrigerated circulating bath AD07R (PolyScience, Niles, IL, USA) was used to keep a constant temperature of 293 K during the whole time (2 h) of the synthesis process. In the next step, to remove the remaining substrates, the obtained gel was rinsed by using pure ethanol and centrifuged. Finally, SbSI gel was inserted in a vacuum chamber under reduced pressure at room temperature in order to evaporate ethanol from its volume. The SbSI nanowires in prepared xerogel have cross dimensions 10 nm to 50 nm and length of several microns. Further information on the sonochemical process, as well as on obtained SbSI xerogel (e.g., SEM and HRTEM micrographs, SAED and XRD patterns, DRS spectrum) in [34].

The prepared nanowires have been used to produce CSNC. In this case, SbSI nanowires have been mixed with pieces of Northern Bleached Softwood Kraft (NBSK) cellulose fibers in the mass ratio of 1:4. Ultrasounds were used again to guarantee a homogeneous mixture of both components. The dilute suspension of CSNC was applied on blotting paper and compressed to obtain a sheet of randomly interwoven cellulose fibers and SbSI nanowires. The samples of CSNC were then cut from so prepared sheet. Gold electrodes were sputtered on opposite sides of the samples by using Sputter coater Quorum Q150T ES (Quorum Technologies Ltd., Laughton, UK). The copper wires were coupled with electrodes by High Purity Silver Paste 05002-AB (SPI Supplies, West Chester, PA, USA). The samples were covered with silicones rubber (Elastosil N10 from DRAWIN Vertriebs-GmbH, Hohenbrunn, Germany) to guard them against the ambient moisture. Further details of the process, the composite morphology, and other results for CSNC, see [29].

To produce an ESNC the SbSI nanowires and LH288 epoxy resin (HAVEL COMPOSITES, Svesedlice, Czech Republic) were mixed in 20% and 40% mass ratio. Then, they were pre-mixed mechanically and then mixed again in ultrasound by using the same set-up configuration as described above. At this stage, the preparation of the ESNC sample and FRP laminate diverged.

In the case of the ESNC sample, the hardener H281 (HAVEL COMPOSITES, Svesedlice, Czech Republic) was added with a volume proportion to resin 1:4, according to technical requirements. A thin layer of so prepared mixture was placed on a glass substrate and left in an Environmental Chamber SH-242 (Espec, Osaka, Japan) at a constant temperature (283 K) for 24 h to be cured. Part of the mixture was also placed in the same chamber between two electrodes to enable the alignment of SbSI in the electric field. The applied electric field intensity was 10 kV/cm. After the main curing, the same procedure as in for CSNC was applied to produce a nanogenerator. More details of the process, the composite morphology, and other results for 20% CSNC, one can find in [26].

The sample surface dimensions of ESNC and CSNC were the same and equaled 10 × 10 mm. Sample thicknesses were 0.10 mm and 0.05 mm for ESNC and CSNC, respectively.

To produce the FRP laminate, 10 layers of a plain-woven glass fabric (KROSGLASS, Krosno, Poland) were used as a reinforcement. After preparing the mixture of SbSI nanowires with the resin, the same hardener was added to it in the same ratio as mentioned above. The whole was mixed mechanically. The silver-conducting paint (05002-AB, SPI Supplies, West Chester, PA, USA) was applied as electrodes on two glass fabric cuttings. Copper wires were connected to both electrodes to provide an electrical signal outside of the sensor. Then, the active material (a mixture of epoxy resin, SbSI nanowires, and hardener) was applied onto the electrodes and one piece of glass fabric was placed between them, and a mixture was penetrated throughout this layer. It allows avoiding a short circuit of the electrodes in further process. The material was left for one hour to pre-cure. The cured mixture is, in fact, an ESNC. Next, the prepared sensor was placed between the remaining plain-woven glass and copper wires were pulled out of a stack. The prepared stack of glass layers was placed into a preform, that was filled with the same resin and hardener by using the VARTM (vacuum-assisted resin transfer molding) method. After curing, the plates including the active layer of ESNC were cut mechanically into the samples. A more detailed description of the FRP laminate preparation process is described in [31].

Sample photographs and SEM images were taken by a Stemi 2000-C stereoscopic microscope (Carl Zeiss, Oberkochen, Germany) equipped with an Olympus DP25 camera and a Phenom PRO X scanning electron microscope (Phenom World, Eindhoven, Netherlands), respectively.

Piezoelectric response of CSNC and ESNC were measured in air at the temperature of 293 K. The sample was mounted on a plate made of plexiglass that was attached to a vibration generator by a wax thin layer. The vibration amplitude and frequency were controlled by using vibrometer WH-30 with octave filter FO-1 (STANMARK Products, Cracow, Poland). The U–I characteristics were registered for various load resistances by using the Zeal resistance box (1 Ω–10^9^ Ω). The output voltage was acquired by EG & G 5110 amplifier (Princeton Applied Research, Oak Ridge, TN, USA). The values of load resistance were recalculated considering its input impedance of 100 MΩ.

The piezoelectric properties of the FRP laminate sensors were determined by 3-point non-destructive (within the elastic deflection range) bending tests. The tests were performed by the INSTRON 4469 machine (INSTRON, Norwood, MA, USA). Spacing between supports was 200 mm. The sample was bent to a deflection of 1.0 mm at various speeds of loading bar movement (1, 2, 5, 10, 20, 40, 60, 80, 100, 150, and 300 mm/min). Then, the sample was left under deflection for 120 s and reloaded at the same speed. After 30 s (time necessary to sensor discharge), the load–reload cycle was executed for the next loading speed. The electric signals of FRP laminate were registered by Keithley 6517A electrometer (Keithley Instruments, Cleveland, OH, USA). During the nondestructive deflection, the load–deflection curves were recorded.

## 3. Results and Discussion

**Figure 2:**
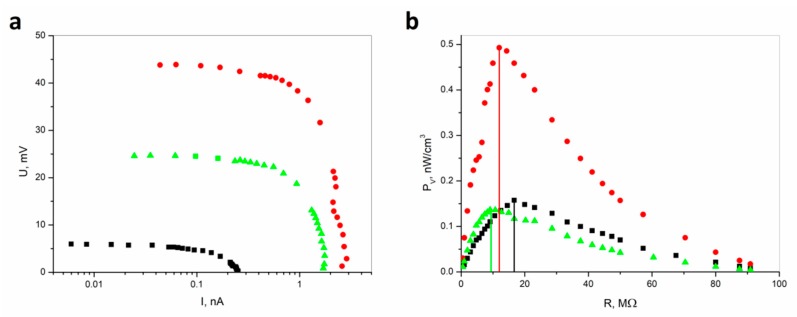
(**a**) Comparison of U–I characteristics and (**b**) volume power density (P_V_) recorded under vibration excitation (f = 24 Hz, A = 1 mm) for different load resistances for CSNC (■) and for ESNC with 20% (●) and 40% (▲) concentration of SbSI nanowires. The vertical lines indicate the values of sample resistance corresponding to the power maximum (description in the text).

Figure 2 shows the comparison of U–I characteristics under vibration excitation for different load resistances for CSNC and ESNC composite.

**Figure 3:**
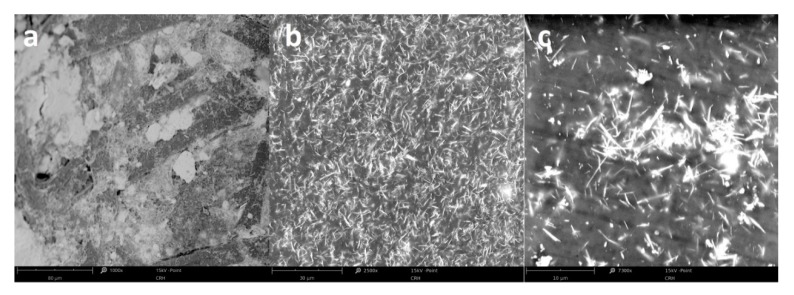
SEM micrographs of CSNC (**a**) and ESNC with 20% (**b**) and 40% (**c**) weight concentration of SbSI nanowires.

One can see that for CSNC registered voltages and currents have lower values than for ESNC. The open-circuit voltage reaches only 6 mV for the CSNC. In the case of the ESNC, it is 45 mV and 25 mV for a composite with 20% and 40% concentration of SbSI nanowires, respectively. Due to miscellaneous sample thickness, the power has been recalculated based on measured U–I characteristics. Then considering the sample volume the volume power density (P_V_) vs. load resistance (R) curves were determined. Despite smaller voltage and current values for CSNC, the determined P_V_ is comparable with 40% ESNC, due to a fact that the CSNC sample is thinner than the ESNC sample. The maximum volume power densities reach values of 0.157 nW/cm^3^, 0.492 nW/cm^3^, and 0.136 nW/cm^3^ for CSNC, 20% ESNC, and 40% ESNC, respectively. In the case of the 40% ECNS sample, the power density is nearly 4 times lower than for 20% ECNS due to the conglomeration of SbSI nanowires. This is well known; the maximum P_V_ is reached for load resistance and equals the internal resistance of the sample. Thus, determined internal sample resistances are 16.6(33) MΩ, 12.0(24) MΩ, and 9.1(16) MΩ for CSNC, 20% ESNC, and 40% ESNC, respectively. It must be underlined that the root-mean-square voltage values were measured, as well as U–I characteristics were acquired in real conditions (load resistance), considering the measurement setups. However, it is possible to enlarge the received power during vibration by applying external load mass on the sample [26]. One can notice that an increase of SbSI nanowires concentration leads to a decrease in internal resistance. This is because SbSI nanowires have lower resistance than the matrix. On the other hand, they are more homogeneously distributed throughout the composite which leads to more efficient power generation. One can see that there is also an influence of matrix material for the internal resistance of the samples. These explanations are supported by SEM images presented in Figure 3 and discussed below.

Figure 3 shows the morphology of CSNC and ESNC with different concentrations of SbSI nanowires. Due to high difference in SbSI nanowires (lateral dimensions 10–100 nm and lengths up to several µm [34]), and cellulose fibers (diameter 10–30 µm and length up to 1 mm [35,36]) dimensions, the SbSI nanowires conglomerate within cellulose fiber matrix (Figure 3a). In the case of ESNC with 20% weight concentration (Figure 3b), there is no agglomeration of SbSI nanowires and they are randomly dispersed in the epoxy resin matrix, forming so-called 0–3 composite [37]. With the increase of SbSI concentration, they become agglomerate (Figure 3c). Similar dependence has been noticed also in the case of examined FRP laminate and will be discussed further below.

**Figure 4:**
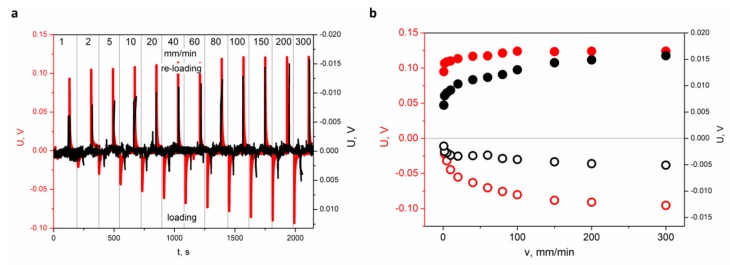
(**a**) Piezoelectric responses generated by the sensor recorded for different loading velocity (1, 2, 5, 10, 20, 40, 60, 80, 100, 150, and 300 mm/min) at constant deflection value x = 1 mm for FRP laminate with 20% (red curve, left scale) and 40% (black curve, right scale) of SbSI nanowires weight concentration. (**b**) Determined values of maximum voltage for different speed of deformation and constant value of maximum deflection (x = 1 mm) during loading (○—20%, ○—40%) and reloading (●—20%, ●—40%), respectively. Left and right scales refer to FRP laminate with 20% and 40% of SbSI nanowires weight concentration, respectively. The sensor was reloaded at the same speed as at loading.

Figure 4 presents the results of the piezoelectric response registered during the bending tests of the FRP laminate sample containing the sensor with different concentrations of SbSI nanowires.

**Figure 5:**
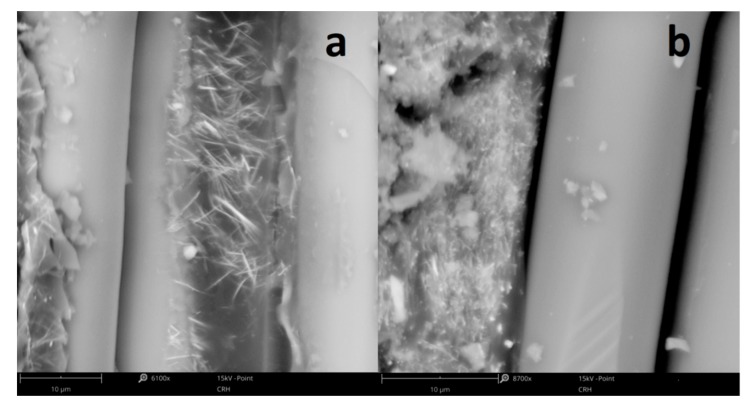
SEM micrographs of FRP laminate with (**a**) 20% and (**b**) 40% of SbSI nanowires weight concentration showing the boundary between the epoxy resin-SbSI nanowires composite and glass-fiber reinforcement.

One can see that the piezoelectric response of the FRP laminate sample with 20% of SbSI nanowires weight concentration is nearly one order of magnitude higher than for the one with 40% of SbSI nanowires concentration (Figure 4). However, the character of changes is the same in both cases. The FRP sensor containing 40% weight concentration of SbSI nanowires shows weaker piezoelectric activity than the sensor containing 20% SbSI nanowires. This seems to be strange at first glance. However, the obtained results leave no doubt in this matter as for ESNC. The explanation for this effect can be found in the local agglomeration of the nano-additive in the resin matrix. The mechanism of piezoelectric charge generation for FRP laminate has been described in [31], and the differences can be explained taking into consideration the morphology of both sensors (Figure 5).

**Figure 6:**
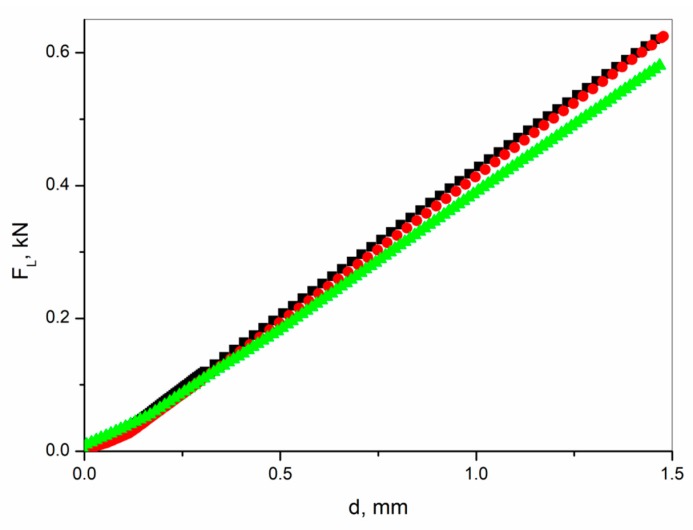
Load force vs. deflection curves recorded for the FRP laminate without an active layer (■) and with an active layer containing integrated sensor-area made of epoxy resin with 20% (●) and 40% (▲) weight concentration of SbSI nanowires.

Here, as well as in the case of ESNC, one can see the agglomeration of SbSI nanowires in FRP sample with 40% of SbSI nanowires weight concentration (Figure 5b), which does not appear for 20% concentration (Figure 5a). It is most likely due to the increased probability of mutual mechanical tacking of individual nanowires during the mixing process, and then joining subsequent nanowires with the resulting agglomerate. This is an effect often found in composites containing nano-dimensional components. Similar phenomena have been previously noticed for carbon nanotubes [38,39]. In those articles, certain optimum content of the nano-additive (carbon nanotubes) was found at which material properties (electrical conductivity, modulus of elasticity) achieved a maximal value. Below and over the optimal concentration, values of these parameters began to fall. The authors explained such an effect, among other issues, by a local agglomeration of the nanomaterial. As well here, with an increase of SbSI nanowires weight concentration, they more likely tend to conglomeration. In this case, agglomerated nanowires are surrounded by a nonconductive epoxy resin matrix. The conglomeration of SbSI nanowires means that they are less randomly dispersed in a matrix, and then a piezoelectric charge is not uniformly discharged to electrodes through nonconductive epoxy resin (or cellulose) matrix. The piezoelectric response then becomes smaller in both cases of ESNC and FRP laminate. Moreover, the increase in SbSI nanowires content results in a decrease in the mechanical response of the composite. Figure 6 presents the load–deflection curves for the nondestructive deflection tests up to 1.5 mm.

Deflection curves (Figure 6) for laminate containing 20% of SbSI nanowires and without the integrated sensor overlap. One can see that further addition of SbSI nanowires (40% weight in this case) results in the weakening of the laminate. The same deflection is reached at a lower load force. After performing nondestructive bending research, the samples were subjected to regular static bending tests. Also, in this case, the FRP laminate containing 40% of SbSI nanowires was the weakest one. Determined values of flexural strength were 393(19) MPa, 381(13) MPa, and 314(11) MPa, whereas values of flexural modulus were 19.9(5) GPa, 19.7(6) GPa, and 19.2(1) GPa, for FRP without an active layer, and with an active layer containing 20% and 40% weight of SbSI nanowires, respectively. Laminate integrated sensors find an application in aviation, construction monitoring, etc. Therefore, this undesirable effect will not limit the application of these structures in contradistinction to, e.g., metal–fiber laminates (FML) [40].

**Figure 7:**
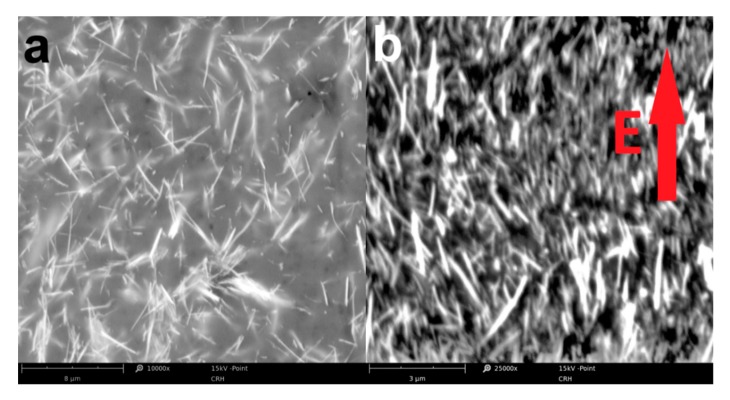
Cross-section SEM micrographs of ESNC with 40% weight concentration of SbSI nanowires cured without (**a**) and under (**b**) electric field with an intensity of 10 kV/cm. The arrow represents the direction of the external electric field applied during the curing process.

One of the possible solutions to improve the piezoelectric properties of ESNC is the application of an electric field for the duration of the curing process. Figure 7 presents SEM images of ESNC cured without and with the applied electric field.

One can see (Figure 7b), all nanowires are aligned, and its c-axis is nearly parallel to the direction of the electric field. Recall that the curing process was carried out in T = 283 K. This temperature is lower than the Curie temperature of SbSI nanowires (T_C_ = 292(1) K) [34]. Therefore, SbSI nanowires are in the ferroelectric phase and align along the external electric field. However, not all of them aligned perfectly along external electric field direction, even though the resin with low viscosity was used (500–900 mPa·s at room temperature according to the manufacturer). The biggest value of the deflection angle of single nanowire c-axis from external electric field direction is less than 35°, whereas its medium value is 12.5(39)°. Note that the longer the nanowire is, the higher the deflection angle forms. This is due to the increase of resin viscous torque acting on the nanowire, which is not overcome by electric field torque rotating the long nanowire. Except for SbSI nanowire alignment, no conglomeration of them is visible on the SEM micrograph (Figure 7b). The surface charges that may be constrained on ferroelectric domain boundaries [41,42] in SbSI nanowires may lead to their separation in the matrix material. The 0-3 type composite simultaneously rebuilt into the 1-3 type composite [37]. Possibility of ferroelectric SbSI nanowires alignment in the external electric field is a well-known phenomenon recently studied [43,44] and occurs spontaneously, i.e., while fabricating composite by electrospinning method [28].

The question might arise about the working temperature range of the presented nanocomposites. According to the datasheet provided by the producer, cured LH288 resin has a maximum long-term operating temperature of 353 K. This temperature corresponds approximately to the glass transition temperature of the resin. The safe temperature of permanent use of the resin should not exceed 323 K for working as an independent material (without significant filling, e.g., with fiberglass), whereas the flash point of the resin is above 423 K. During the analysis of the curing process, no influence of SbSI on the intensity of this process was observed. The thermal decomposition of SbSI begins in 545 K. Above 609 K the remaining material is Sb_2_S_3_ (see the thermal gravimetry analysis (TGA) in an appendix of [45]). Thermal decomposition of SbSI reaches a higher temperature than the working temperature range of epoxy resin; therefore, the ESNC is applicable in the whole operating temperature range of epoxy resin. In the case of CSNC, the temperature limit is set by SbSI, whereas thermal decomposition of cellulose occurs at ~573–673 K (see TGA curves in [46,47]), which is just a little higher than the thermal decomposition temperature of SbSI. Moreover, the study of thermal decomposition of epoxy resin (of another type than in this article) and NBSK cellulose (the same type of cellulose as in this article) composite is shown in [47]. The authors state that the incorporation of cellulose filler enhanced the thermal stability of all epoxy nanocomposites compared to pure epoxy composites [47]. One can expect that the CSNC application will be limited by a lower SbSI decomposition temperature of 545 K.

## 4. Conclusions

Comparison of piezoelectric responses registered for vibration excitation (f = 24 Hz, A = 1mm) for different load resistances for CSNC and for ESNC with 20% and 40% concentration of SbSI nanowires has been presented. It has been shown that the matrix material has had a significant influence on the piezoelectric properties of the composite. Moreover, a comparison of ESNC for different nano-additive leads to a conclusion that an increase of a volume fraction of the nano-piezoelectric material (SbSI nanowires) leads to decreasing of the output signal and power. It is influenced by the agglomeration of SbSI nanowires in the matrix material. Note that agglomeration may take place even for a smaller amount of nanowires but the increase of its concentration results in a greater quantity of agglomerates. More detailed studies on this phenomenon for ESNC will be performed in the future.

Agglomeration occurs also in the case of FRP laminate (Figure 5). Moreover, the increase in SbSI nanowires mass fraction from 20% to 40% causes an evident decrease in FRP laminate mechanical response signal (Figure 6). Therefore, further examination for FRP laminate is also necessary to find the best concentration of SbSI nanowires in a sensor to achieve the highest piezoelectric properties and mechanical strength of the composite.

Preliminarily conducted research shows that the application of the external electric field allows SbSI nanowires aligning which should improve the piezoelectric response of the composite due to anisotropy of piezoelectric modulus and electromechanical coefficient of SbSI nanowires. Besides, it can also be one of a possible solution to the conglomeration issue.

Summarizing, for the first time, piezoelectric response for vibration and its comparison for CSNC and ESNC with different SbSI nanowires concentration has been presented. The discussion presented above leads to the conclusion that both composites are appropriate to work in ambient temperatures. However, due to phase transition in SbSI, the highest piezoelectric response and performance of the samples are achieved in lower temperatures. The influence of SbSI nanowires concentration for the conglomeration of them in ESNC and FRP laminate has been presented for the first time, and the solution to this issue has been proposed. Moreover, the thinner sample preparation and curing the resin under the applied electric field will result in more efficient power generation. Taking into consideration the discussion presented above and the mechanical properties of the ESNC and CSNC composites, it leads to the conclusion that ESNC is a promising material to harvest vibration energy and CSNC may be used as smart wearable textiles, in the future.
